# Enhanced Dynamic Impact Resistance of 3D-Printed Continuous Optical Fiber-Reinforced Helicoidal Polylactic Acid Composites

**DOI:** 10.3390/polym15234599

**Published:** 2023-12-01

**Authors:** Aiqiu Wang, Ying Liu, Rui Yan, Yuye Wang, Pengjun Luo, Yangbo Li

**Affiliations:** College of Hydraulic and Environmental Engineering, China Three Gorges University, Yichang 443002, China; 202108150021028@ctgu.edu.cn (A.W.); 20091827@ceic.com (Y.L.); yanrui0591@foxmail.com (R.Y.); y13635287808@163.com (Y.W.); luopengjun233@gmail.com (P.L.)

**Keywords:** high-strain-rate impact, COF-HP, helicoidal Polylactic Acid (HP), SHPB

## Abstract

Characterized by light weight and high strength, composites are widely used as protective materials in dynamic impact loading under extreme conditions, such as high strain rates. Therefore, based on the excellent tensile properties of continuous fiber and the good flexibility and toughness of the bionic spiral structure, this study uses a multi-material 3D printer to incorporate continuous fiber, and then modifies the G-CODE file to control the printing path to achieve the production of a continuous fiber-reinforced Polylactic Acid composite helicoidal (spiral angle 60°) structure (COF-HP). Dynamic behavior under high-strain-rate impact experiments have been conducted using the Split Hopkinson Pressure Bar (SHPB). Stress–strain curves, impact energy curves and high-speed camera photographs with different strain rates at 680 $s^{-1}$ and 890 $s^{-1}$ have been analyzed to explore the dynamic process and illustrate the damage evolution. In addition, some detailed simulation models considering the incorporation of continuous optical fiber (COF) and different strain rates have been established and verified for deeper investigations. The results show that the COF does enhance the impact resistance of the laminates. When the porosity is reduced, the maximum stress of the continuous fiber-reinforced composite material is 4~7% higher than that of the pure PLA material. Our findings here expand the application of COF and provide a new method for designing protective materials, which have broad application prospects in the aerospace and automotive industries.

## 1. Introduction

**Figure 1:**
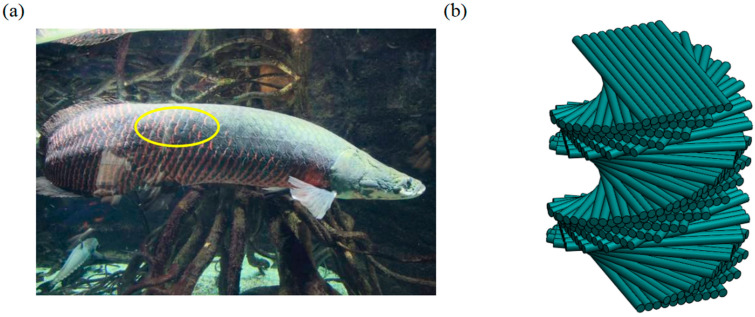
Helicoidal structures in nature. (**a**) The scales of Arapaima [11]. (**b**) The design of the helicoidal structure.

Lightweight and reliable protective materials/structures play an important role in protection under extreme conditions such as high strain rates [1,2], high temperatures [3], high pressures [4], and ultra-high velocity impacts [5,6]. How to design new materials and new structures to resist impact damage in extreme environments is a problem which needs to be solved at present. Inspired by nature, biostructures already have a variety of high performances [7,8,9,10]. Zimmermann et al. [11] have found the scales of Arapaima (Figure 1a) have an obvious lamellar arrangement, which can resist the predation of piranhas. The elytra of the beetle have different fiber arrangements in different life cycles [12,13]. The toe bar of the Odontodactylus Scyllarus [14] consists of several stacked toe bar structures that provide toughness and avoid damage during attack. These animals all have the same structure, a twisted laminate structure, consisting of unidirectional fiber layers with a small misfit angle between the fiber layers, resulting in a 180° helicoidal rotation along the vertical axis of the stacking plane, as shown in Figure 1b. Previous studies have shown that the optimal helicoidal angle is 60° under high-strain-rate impact [15].

Currently, fiber-reinforced composites [16,17,18,19,20,21,22] have been investigated widely due to good recyclability and low cost. The mechanical behavior of carbon/glass fiber-reinforced composites has already been discovered [23,24,25,26,27,28,29]. Flax fiber-reinforced composites [20,30] also have become a hot topic because of their ultra-high specific stiffness. Optical fibers are widely known for thermometric conduction [31,32], and have stronger tensile and bending strengths, while the behavior of COF-HP has not been studied clearly. Rui Yan et al. [33] have found that the COF can appropriately improve the tensile strength, toughness and ductility, and the bending strength is increased by 10–20%. Previous experiments and investigations only focused on the typical mechanical behavior, while not considering their dynamic behavior under high-strain-rate impact, which is of great significance for the selection of protective materials.

Fused deposition modeling(FDM) is a type of deposition molding technology [34] and the FDM 3D printer can customize the path [35,36], which is good for us in the fabrication of the helicoidal laminates. Rui Yan et al. [33] have designed an FFF printer that can realize simultaneous printing of Polylactic Acid (PLA) and COF, which has solved the problem of bonding between COF and PLA effectively. This also provides an idea and method for incorporating COF into PLA. Due to the accuracy of the 3D printer not being precise, there are some pores in these specimens, which weaken the mechanical properties of the specimens. There are few studies on the effect of the existence of pores on the mechanical properties of 3D printed specimens. Yin et al. [37] have found that the presence of pores can enhance the toughness of the specimen.

## 2. Materials and Methods

In order to explore the impact resistance of COF-HP at high strain rates, we have used the self-developed multi-material continuous fiber 3D printer to prepare a series of specimens with the helicoidal angle of 60°. Some measures have also been adopted to compact these specimens to further explore the effect of voids on the structures. For the treated specimens, the SHPB impact testing under high strain rate has been carried out to investigate their impact resistance. A high-speed camera has been used to capture the dynamic impact photos, and DIC-1.4.0.1085.20220714 software has been utilized to analyze the deformation and strain. In addition, in order to verify the reliability of the test results, the ABAQUS platform [38] for computation has been conducted to analyze the destruction forms and verify the accuracy of the dynamic impact loading experiments.

### 2.1. Fabrication of Material and Specimens

**Figure 2:**
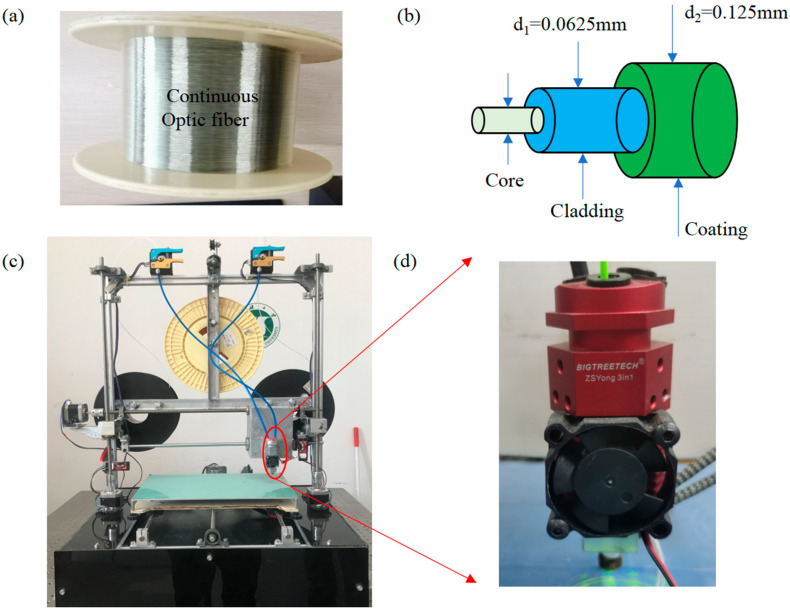
Specimen forming materials and printers. (**a**) The macrostructure of COF. (**b**) The schematic of COF. (**c**) Self-developed multi-material continuous fiber 3D printer. (**d**) Three-in and one-out nozzle.

The PLA material used in the experiment is the product provided by Shenzhen JGMAKER Co., LTD. According to the parameters provided by the processor, the processing temperature of PLA is between 195 °C and 230 °C, and the temperature of 200 °C has a relatively good machinability. The selected COF—Figure 2a—is made of glass, which consists of a central core, cladding, and outer coating, as shown in Figure 2b. According to the parameters provided by the manufacturer Guangdong Fengda photoelectric technology Co., LTD, the outer diameter, axial tensile strength and bending strength are 0.125 mm, 2000–3000 MPa, and 175 MPa, respectively. The central core of COF is made of high-purity silica glass, the cladding is composed of silica with a small amount of dopant, and the outer coating is composed of acrylate, silicone rubber and nylon. The specimens are fabricated by a self-developed multi-material continuous fiber 3D printer, as shown in Figure 2c. There is a three-in and one-out nozzle (Figure 2d) in the 3D printer, which can print the COF-HP. And the printing method is the same as in Rui Yan et al. [33], that is, the combination of PLA-COF-PLA feeding is adopted, and two PLA filaments wrap an optical fiber to ensure that the continuous optical fiber is in the center of the PLA filament when the material is extruded.

The 3D printer was used to prepare several groups of cylindrical specimens with a spiral angle of 60° and a diameter of 16 mm. Through the printing test, the relevant parameters of the best printing effect were obtained: nozzle temperature 210 °C, printing speed 30 mm/s, off-line setting of the outer profile circle number 2, and filling rate 100%. We selected the way of linear filling, used the machine matching slicing software to export the path file, modified the G-CODE file to change the coordinates of the print path, and realized the printing of the target specimen. In addition, the original diameter of the PLA material was 1.75 mm. Although the diameter of the printer nozzle was 1.4 mm, the diameter of the PLA filament after printing would be slightly larger than the diameter of the nozzle.

**Figure 3:**
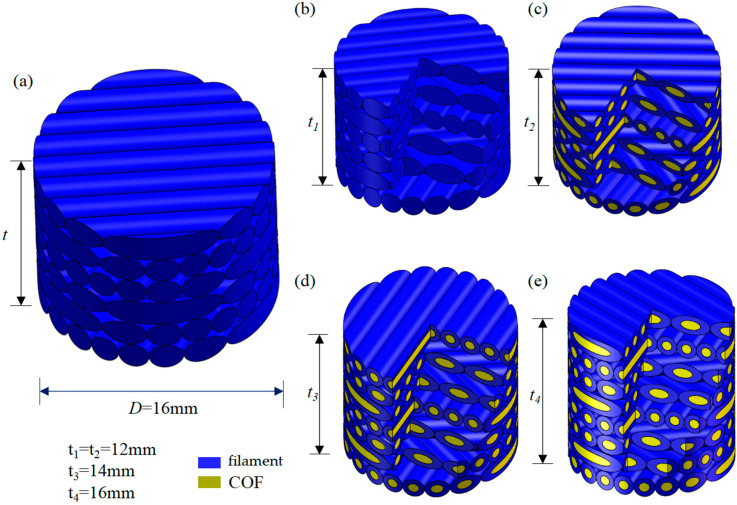
The description of specimens. The cylinders feature the diameter *D* and the height *t*. (**a**) The outline of the specimen. (**b**) 6-layer HP. (**c**) 6-layer COF-HP. (**d**) 7-layer COF-HP. (**e**) 8-layer COF-HP.

According to different exploration purposes, relevant variables were introduced to make specimens. In order to explore the enhancement effect of continuous optical fiber (COF), it is necessary to make specimens with or without the addition of COF as a variable. We prepared 6 layers of helicoidal Polylactic Acid (6-Layer HP) and 6 layers of continuous optical fiber-reinforced helicoidal Polylactic Acid (6-layer COF-HP), as shown in Figure 3b,c. In order to explore the impact of the porosity introduced by the incorporation of COF on the impact resistance of the test pieces, different porosity between the test pieces should be taken as a variable. Therefore, we prepared 7 layers of continuous optical fiber-reinforced helicoidal Polylactic Acid (7-layer COF-HP) and 8 layers of continuous optical fiber-reinforced helicoidal Polylactic Acid (8-layer COF-HP), as shown in Figure 3d,e. When the printed specimens with different layers are compressed to the same height, specimens with the same structure and appearance size but different porosity are obtained. Using the characteristics of different melting points of PLA and optical fiber, the pores in the specimen were eliminated. The printed specimen was put into a ring mold with an inner diameter of 16 mm and a height of 10 mm, and then put into an oven at 70 °C–80 °C until the PLA softened to the glass state. The internal structure of the specimen was not damaged. The specimens were subjected to a quasi-static compression test under static load of low strain rate and were compressed to a height of 10 mm. By calculating the changes mentioned before and after compression of the specimen, we can calculate the approximate change of pore content. In this process, the specimen is compacted so that the contact between the fiber and PLA is closer. The compaction under the action of low strain rate will eliminate the defects such as micro-cracks and micro-pores generated during the preparation of the specimen, without destroying the original helical structure of the specimen. We called these compacted specimens the following: 6 layers of compacted helicoidal Polylactic Acid (6-layer CHP), 6 layers of compacted continuous optical fiber-reinforced helicoidal Polylactic Acid (6-layer COF-CHP), 7 layers of compacted continuous optical fiber-reinforced helicoidal Polylactic Acid (7-layer COF-CHP), and 8 layers of compacted continuous optical fiber-reinforced helicoidal Polylactic Acid (8-layer COF-CHP). Table 1 provides a more intuitive understanding of the differences in process parameters between specimens.

### 2.2. High-Strain-Rate Impact Testing

**Figure 4:**
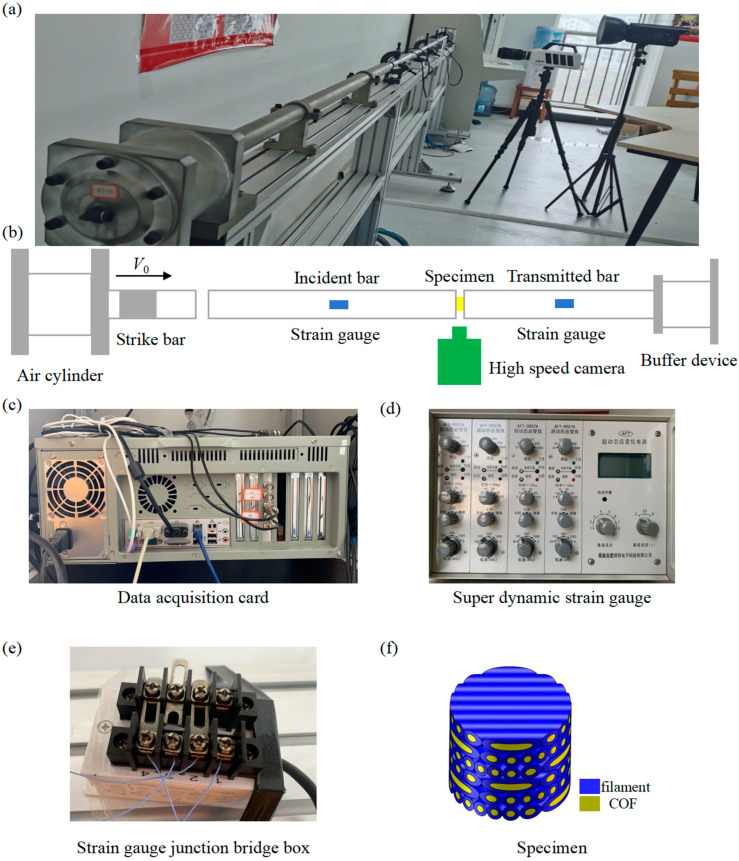
SHPB testing system used for dynamic compressive loading under high rate impact. (**a**) Experimental set-up. (**b**) Schematic of SHPB testing system. (**c**) Data acquisition card. (**d**) Super dynamic strain gauge. (**e**) Strain gauge junction bridge box. (**f**) Schematic of the specimen.

Dynamic tests under different high strain rates for 680 $s^{-1}$ and 890 $s^{-1}$ have been carried out by the SHPB system, which is a compression type, as shown in Figure 4a. In this experiment, the length of the strike bar, incident bar and transmitted bar are 350 mm, 1500 mm and 1200 mm, respectively, and with a diameter of 30 mm. The elastic modulus, elastic wave velocity, and density are *E* = 70 GPa, *v* = 5000 m/s and *ρ* = 2800 kg/m^3^, respectively. The strain junction bridge box (Figure 4e) transmits the strain signal which comes from the strain gauges pasted on the incident bar and transmitted bar to the super dynamic strain gauge (Figure 4d), and the stress–stain curves are obtained through the data acquisition card (Figure 4c).

**Figure 5:**
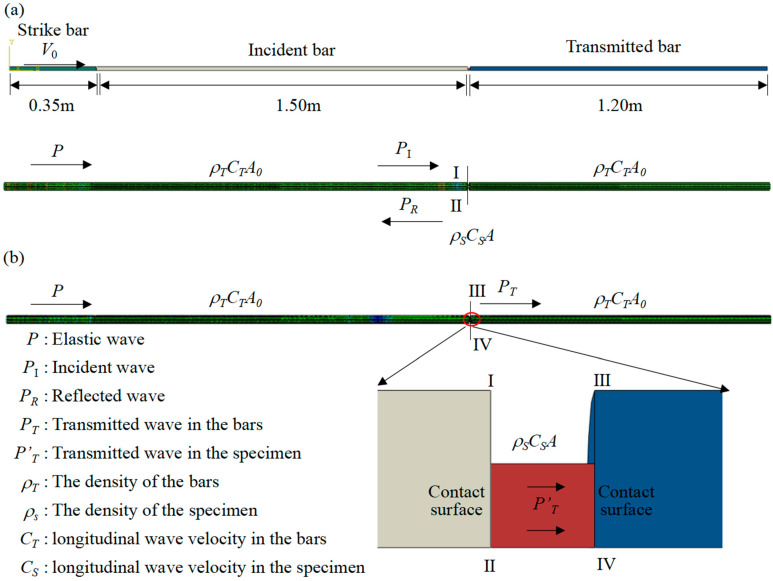
The overall transmission process of the stress wave. (**a**) Propagation of stress waves in the incident bar and specimen. (**b**) Propagation of stress waves in specimen and transmitted bar.

The specimen is sandwiched between the incident bar and the transmitted bar. When the strike bar is emitted from the air cylinder and hits the incident bar, elastic waves are generated and the elastic wave propagates from the incident bar to the specimen. When the elastic wave reaches the contact surface between the incident bar and the specimen, a part of the elastic wave is reflected to the incident bar, and the other part propagates through the specimen to the transmitted bar, I-II and III-IV represent the two surfaces in contact with the specimen. Figure 5a,b show the overall propagation process of the elastic waves during the experiments. In addition, the elastic wave propagations within the incident and transmitted bars are recorded by the strain gauges on the two bars, respectively, as shown in Figure 5b. To better observe the tiny deformation of specimens under high-strain-rate impact, a high-speed camera Revealer 5F01 has been used at 5 μs per frame of 1280 × 1024 pixels to capture the dynamic behavior of specimens.

In this experiment, the wave dispersion phenomenon can be ignored due to the large slenderness of the bar. Considering the stress balance inside the specimen $\varepsilon_{i}\left(t\right)+\varepsilon_{r}\left(t\right)=\varepsilon_{t}\left(t\right)$, the stress, strain, and stress–strain curves under different strain rates are all obtained according to the one-dimensional stress wave theory and the assumption of uniformity.

(1) $$\varepsilon =-\frac{2C_{0}}{L_{s}}\int_{0}^{t}\varepsilon_{r}\left(t\right)dt$$

(2) $$\sigma =\frac{EA_{0}}{A}\varepsilon_{t}\left(t\right)\;$$

(3) $$\dot{\varepsilon }=\frac{d\varepsilon }{dt}=-\frac{2C_{0}\varepsilon_{r}}{L_{0}}\;$$

Formulas (1)–(3) are classic two-wave test data processing formulas [39], where $\varepsilon_{i}\left(t\right)$ is the incident strain pulse, $\varepsilon_{r}\left(t\right)$ is the reflected strain pulse, $\varepsilon_{t}\left(t\right)$ is the transmitted strain pulse, ε is the strain of the specimen, $C_{0}$ is the wave velocity, $L_{s}$ is the length of the specimen, E is the elastic modulus of the strike, incident, and transmitted bar, $A_{0}$ is the cross-sectional area of the bars, A is the cross-sectional area of the specimen, and $\dot{\varepsilon }$ is the strain rate of the specimen.

### 2.3. Finite Element Modeling

**Figure 6:**
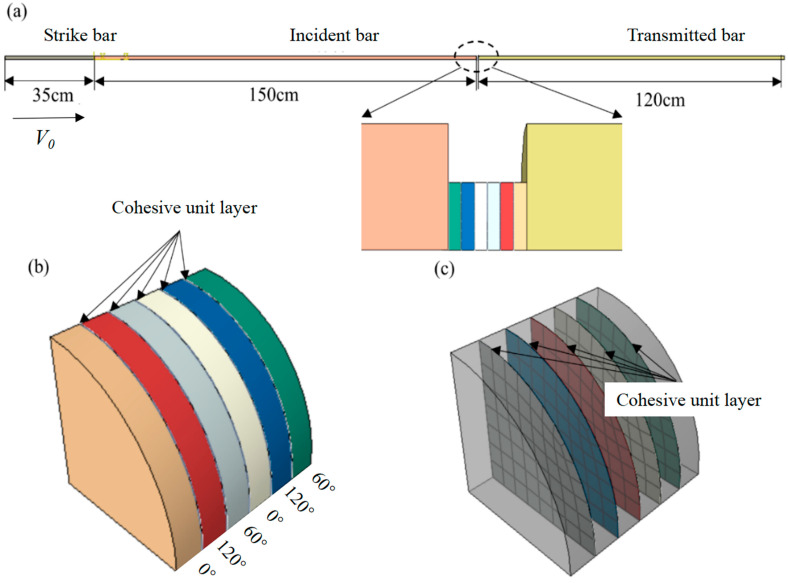
Finite element model of SHPB and continuous fiber reinforced spiral structure. (**a**) Test diagram. (**b**) Specimen stratification and cohesive unit layer. (**c**) The cohesive element layer inside the specimen.

Numerical simulation is used to construct four components: the strike bar, incident bar, spiral structure specimen, and transmission bar. Different material properties are assigned to each component in the attribute module. The strike bar, incident bar and transmission bar mainly have elastic isotropic models. The spiral structure fiber layers are assigned with their respective material properties, inputting engineering constants, and using the three-dimensional Hashin material failure criteria. The assembly module can segment, translate, and rotate the components, and then assemble them according to the test settings. The components are globally divided into meshes, with hexahedral mesh shapes. The mesh type for the compression rod is set as C3D8R (eight-node linear hexahedral element), and the numbers of these bar are 10,500, 45,000 and 36,000, respectively. The mesh type for the spiral structure fiber layers is set as SC8R (eight-node quadrilateral faces with internally continuous shell element), and the material parameters used to simulate the behavior of COF-HP and HP are listed in Table 2. Zero-thickness cohesive elements are added to the contact surfaces of the laminate plates, and the element type is set as COH3D8 (eight-node three-dimensional cohesive element). To prevent mesh penetration during calculations, the mesh size of the compression rod should not exceed 20% of the mesh size between the spiral structure fiber layers. A reference coordinate system is established, and a laminated plate layup model satisfying different working conditions is generated by setting the fiber directions for each layer. In the interaction module, general contact properties are used. In the load and boundary conditions, the initial velocity of the strike bar is set, and all X and Y directions of the incident bar, specimen, and transmission bar are fully fixed. The finite element model created is shown in Figure 6.

## 3. Results and Discussions

### 3.1. Material Mechanical Testing

**Figure 7:**
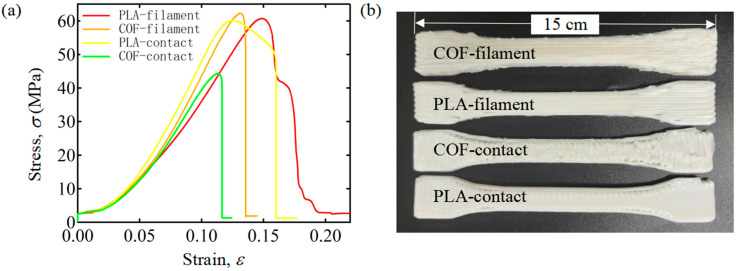
Tensile testing of COF-PLA Composite material and PLA. (**a**) Strain–Stress curves of COF-PLA and PLA under different paths. (**b**) Prepared COF-PLA and PLA standard test pieces.

In order to explore the mechanical properties of COF-HP and HP, we prepared three groups of standard samples with transverse path and filament path, respectively, according to the printing parameters and preparation methods mentioned in Section 2.1. Standard static uniaxial tensile testing of ASTM D638 has been performed on the specimens to obtain the tensile strength. When the printing path is transverse paths, the force direction is perpendicular to the filament direction, and the main test is the contact strength between two PLA filaments. When the printing path is filament paths, the force direction is parallel to the filament direction, and the main test is the tensile strength of PLA filaments. The mechanical testing results are shown in Figure 7a and Table 3. The tensile strength of the contact printing path is shown to be higher than that of the filament printing path by comparing the contact path and the filament path. The tensile strength of PLA has improved, after the COF has been incorporated, from 60.70 MPa to 62.26 MPa. However, the bonding strength decreases from 57.48 MPa to 44.32 MPa, which is caused by the increase in the voids between filament and filament with the addition of COF. As illustrated in Figure 7b, after the incorporation of COF, the space between the PLA filaments increases and is not dense. Therefore, the incorporation of continuous optical fibers will increase the porosity inside the specimen, and the existence of voids greatly reduces the mechanical properties of the specimens.

### 3.2. Porosity Analysis

**Figure 8:**
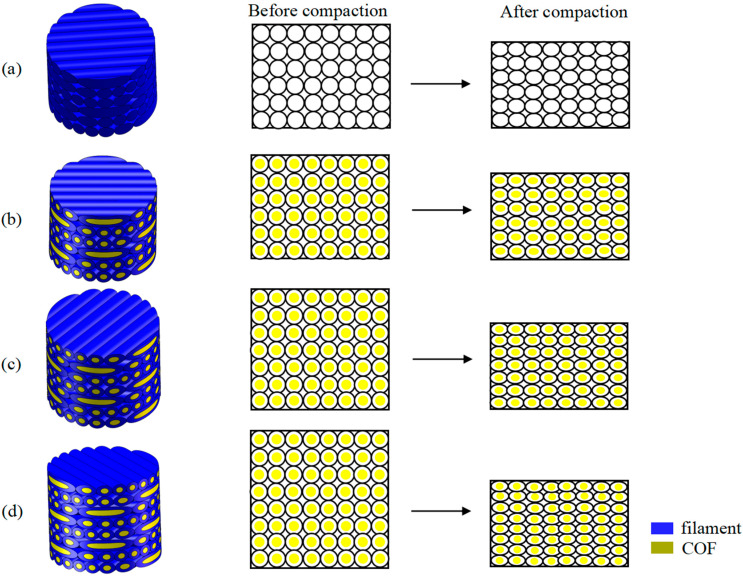
Theoretical models for computation before and after compaction. (**a**) 6−layer HP. (**b**) 6−layer COF−HP. (**c**) 7−layer COF−HP. (**d**) 8−layer COF−HP.

The above studies show that the incorporation of COF increases the porosity inside the specimen. Since the microstructure of the specimen cannot be observed easily, a numerical calculation method has been performed to analyze the internal porosity of these specimens. Figure 8 shows the numerical models: among them, the white ones are PLA filaments, and the yellow ones are COF. Porosity is estimated by calculating the cross section of the specimens. Assuming that the diameter of the filament is 2 mm, the specimens are uniformly deformed after compaction. After calculating, the pore sizes before compaction of the 6-layer specimens, 7-layer specimens and 8-layer specimens are 1.41 mm^2^, 1.97 mm^2^ and 2.20 mm^2^, respectively. After compaction, the pore sizes all decrease to 1.37 mm^2^ by 2.48%, 28.65% and 37.73%, respectively. It is obvious that compaction can indeed reduce the pores inside the specimen. Properly reducing the pores can enhance the dynamic mechanical properties of the composite material, as shown in Figure 8a–c. However, excessive reduction of the pores can reduce the toughness of the composite material and damage the internal structure, as shown in Figure 8d. Appropriate porosity reduction can improve the impact resistance of COF-HP.

### 3.3. Validation

**Figure 9:**
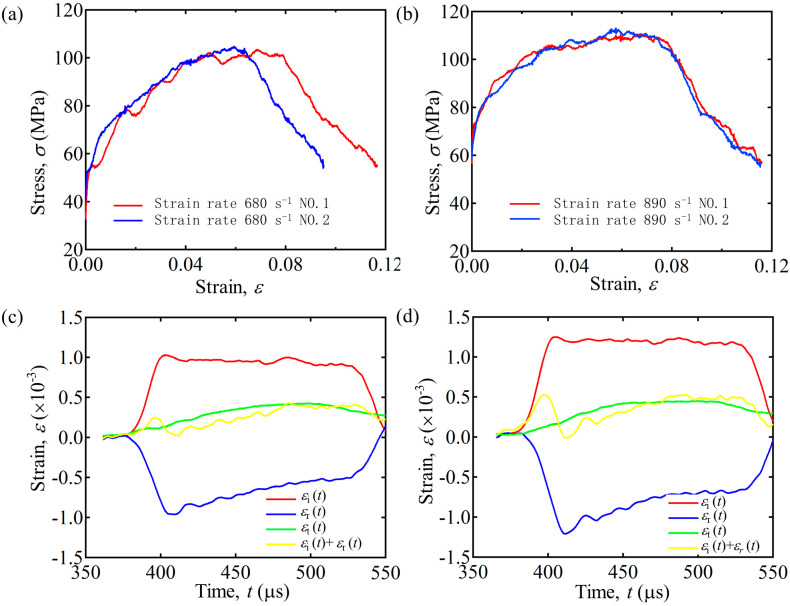
Stress−strain curves of 6−layer HP under impact of different strain rates. (**a**) The strain rate is 680 $s^{-1}$. (**b**) The strain rate is 890 $s^{-1}$. Validation of stress equilibrium for representative SHPB tests of 6−layer HP under different strain rates. (**c**) The strain rate is 680 $s^{-1}$. (**d**) The strain rate is 890 $s^{-1}$.

To ensure the reliability and validity of the test results, it is necessary to verify the stress balance on both sides of the specimen in the SHPB experiments [40]. The 6-layer HP specimen was selected as the sample for the verification test. Some typical tests at different strain rates of 680 $s^{-1}$ and 890 $s^{-1}$ have been enumerated, and they satisfy the stress balance condition ($\varepsilon_{i}\left(t\right)+\varepsilon_{r}\left(t\right)=\varepsilon_{t}\left(t\right)$), as shown in Figure 9c,d. The calculated strain and stress can be obtained at the same time. In the following discussion, we will select a representative stress–strain curve for analysis. In order to confirm the consistency and credibility of the results of the representative curve, we printed more than two groups of samples and conducted the same strain rate test in an experiment; the experimental results showed good repeatability and consistency, as shown in Figure 9a,b.

### 3.4. Impact Resistance Behavior

**Figure 10:**
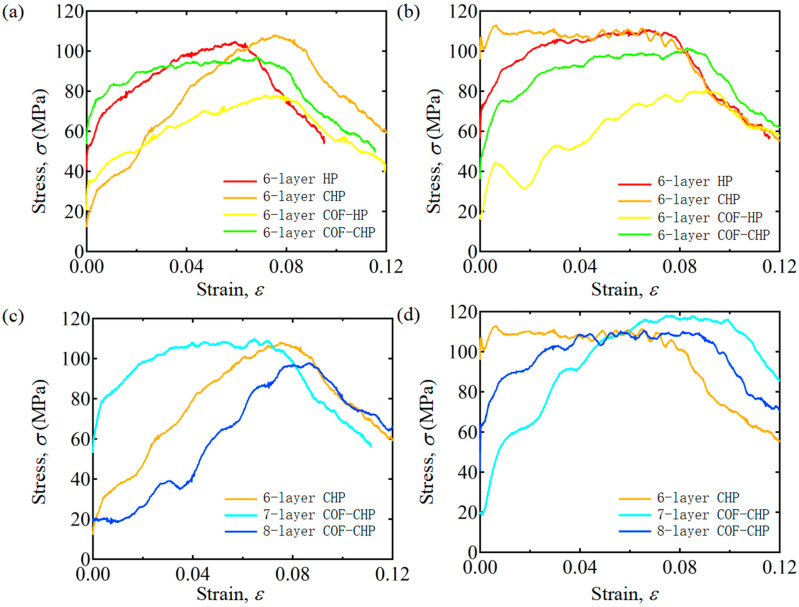
Stress–strain curves under dynamic loading impact with different strain rates. (**a**,**c**) Experimental results with the strain rate of 680 $s^{-1}$. (**b**,**d**) Experimental results with the strain rate of 890 $s^{-1}$.

These impact experiments are conducted by the SHPB system, according to GB 34108-2017 to investigate the reinforcement mechanism of the COF-HP, and the effect of pores on mechanical properties of specimens. Specimens named 6-layer HP, 6-layer COF-HP, 6-layer COF-CHP, 6-layer CHP, 7-layer COF-CHP and 8-layer COF-CHP are used to show impact resistance behavior at different strain rates of 680 $s^{-1}$ and 890 $s^{-1}$, respectively. The strain rate mentioned in the dynamic test refers to the average strain rate during the loading process. And Figure 10 illustrates the dynamic response of specimens.

Figure 10 shows that the incorporation of COF does indeed enhance the impact resistance of the HP when the specimen is sufficiently compacted. Under the action of dynamic impact loading, the stress–strain curves of the specimens can be divided into three stages. The specimen first goes through a linear elastic stage, then rises nonlinearly until it reaches the yield stress, and finally falls. Figure 10a,b show that the incorporation of COF does not significantly enhance the impact resistance of the specimen. The maximum stress σ_max_ = 78.07 MPa of the 6-layer COF-HP specimen is much lower than the maximum stress σ_max_ = 104.84 MPa of the 6-layer HP specimen. However, the dynamic mechanical properties of the specimens have been significantly improved after compaction, and the 6-layer CHP has the best impact resistance, The maximum stress σ_max_ = 109.92 MPa. The voids caused by the incorporation of the COF are compacted and the porosities inside the specimens are reduced. Figure 10c,d show that 7-layer COF-CHP has the best impact resistance and its maximum stress value is greater than that of 6-layer CHP. The incorporation of COF can improve the impact resistance of the specimen under the condition of reducing the porosity. However, the test results of 8-layer COF-CHP are worse than that of 6-CHP. The reason is that in the process of compaction, although the porosity is reduced, its internal structure is also damaged. The 8-layer COF-CHP has been destroyed before the dynamic impact loading.

### 3.5. Strain Rate Effect

In order to accurately characterize the strain rate effect, three representative values from the figure, namely the tangent modulus *E_tan_*, the maximum stress *σ_max_* and the strain *ε* corresponding to the maximum stress when the specimens fail have been listed to describe the experiment, as shown in Table 4 and Table 5. The tangent modulus is defined as the ratio of the stress to the strain at which the specimen yields.

Table 4 and Table 5 show that as the strain rate increases, the *E_tan_* and *σ_max_* of the specimens also increase. The strain hardening phenomenon of COF-HP and HP is obvious. The *E_tan_* and *σ_max_* of the 7-layer COF-CHP are all larger than that of other specimens, which shows 7-layer COF-CHP has better mechanical properties than others. When the strain rate increases from 680 $s^{-1}$ to 890 $s^{-1}$, the *E_tan_* of the 7-layer COF-CHP increases from 1.26 GPa to 1.32 GPa. And the *σ_max_* increases by 9.25% from 108.1 MPa to 118.1 MPa. The larger the *E_tan_* of the 7-layer COF-CHP, the greater the stiffness and the stronger the deformation resistance caused by the dynamic impact loading. With the increase in strain rate, the mechanical properties of COF-HP and HP are improved, and the damage to specimens under dynamic loading is reduced.

### 3.6. Impact Energy Curves

**Figure 11:**
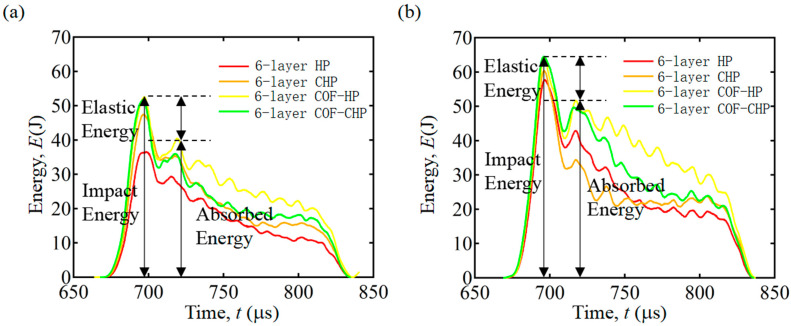
Impact energy curves of 6-layer specimens with different strain rates. (**a**) The strain rate is 680 $s^{-1}$. (**b**) The strain rate is 890 $s^{-1}$.

Impact energy curves have been drawn to explore the change in kinetic energy of the specimens more comprehensively during the experiments. Due to the different quality of the 7-layer and 8-layer specimens, only the 6-layer specimens are compared here. During the dynamic impact process of the experiments, the impact energy is transferred to the incident bar by the strike bar. The impact energy of the incident bar is mainly absorbed through the damage forms such as elastic–plastic deformation, matrix cracking and fiber fracture, and only a small amount is absorbed by friction. Therefore, the impact damage becomes the main energy dissipation mechanism. The transformation and absorption of impact energy can reflect the damage degree of HP and COF-HP, to a certain extent. The impact energy is the sum of the elastic energy and the absorbed energy, as shown in Figure 11. The 6-layer CFO-CHP has the largest impact energy and absorbs the most energy during the impact process, which is consistent with the experimental results. In addition, with the increase in strain rate, the impact energy of the specimens gradually increases, and the absorbed energy of the laminated specimens also increases.

### 3.7. Dynamic Impact Deformation of Specimens

**Figure 12:**
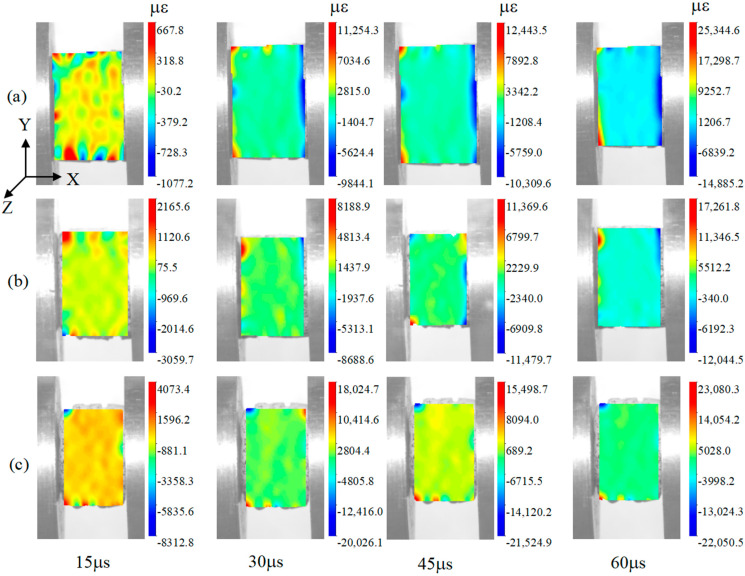
Process of specimen deformations subjected to loading with the strain rate of 680 $s^{-1}$. (**a**) 6−layer HP. (**b**) 6−layer CHP. (**c**) 6−layer COF−HP.

**Figure 13:**
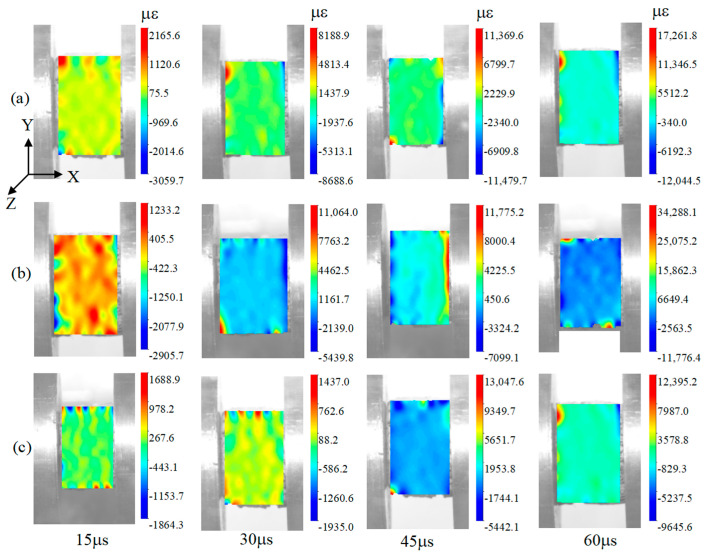
Process of specimen deformations subjected to dynamic loading. (**a**) 6−layer CHP with the strain rate of 680 $s^{-1}$. (**b**) 6−layer CHP with the strain rate of 890 $s^{-1}$. (**c**) 7−layer COF−CHP with the strain rate of 890 $s^{-1}$.

Since the specimen has no obvious external damage, a high-speed camera has been used to photograph the complete process of the dynamic impact loading. The strain at different times has been obtained using DIC-1.4.0.1085.20220714 software. The experimental loading direction is opposite to that of the software, so the values in the heat maps are negative. Figure 12 and Figure 13 show that, with the change in time, the specimens are subjected to dynamic compression, and the amount of compression of the specimens increases with the increase in time. Figure 12 shows that the maximum strains of 6-layer HP, 6-layer CHP and 6-layer COF-HP are 14,885.2 με, 12,044.5 με and 22,050.5 με, respectively. Comparing the strains of the three specimens at the same time, the strain of 6-layer CHP is the smallest and that of 6-layer COF-HP is the largest. It shows that there are pores in the specimens and that compaction enhances the impact deformation resistance of the specimens, while the addition of COF increases the pores inside the specimens.

Figure 12b and Figure 13a illustrate that, with the increase in the strain rate, the strain of the specimen decreases, and the amount of compression deformation is also reduced. The strain of 6-layer CHP decreases from 12,044.5 με to 11,776.4 με, by 2.23%. Figure 13b,c show that the minimum strain of 7-layer COF-CHP is 9645.6 με, which indicates the COF-HP can enhance the performance of composites against impact loading after compaction. The results of the DIC analysis are consistent with the experimental results.

### 3.8. Computational Results

**Figure 14:**
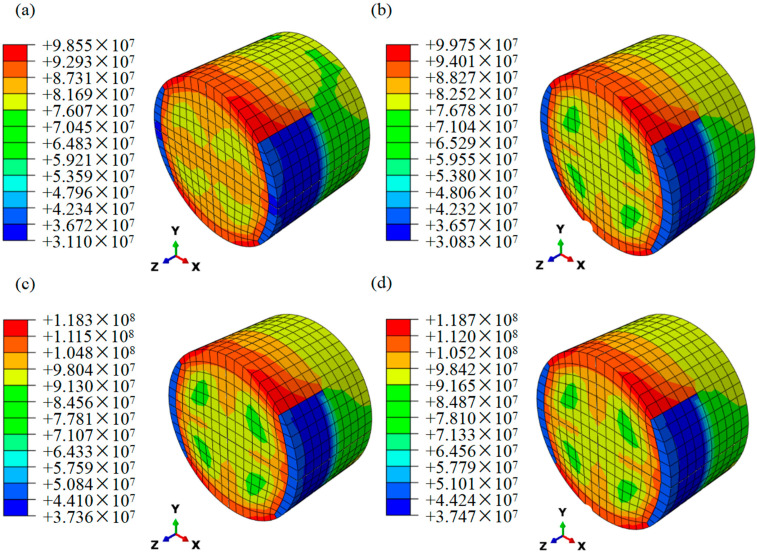
Computational stress applied to different specimens. (**a**) 6−layer HP at strain rate of 680 $s^{-1}$. (**b**) 6−layer CHP at strain rate of 680 $s^{-1}$. (**c**) 6−layer CHP at strain rate of 890 $s^{-1}$. (**d**) 7−layer COF−HP at strain rate of 890 $s^{-1}$.

Figure 14 shows the computational results of the ABAQUS; it is obvious that the maximum stress of the specimens under dynamic impact is consistent with the experimental results. The maximum stress of the specimen appears at the interface between the specimen and the transmitted bar, and at the edge of the cylinder, which indicates the edge of the helicoidal structure is more likely to be damaged under the high-strain-rate dynamic impact loading. The simulation results of 6-layer CHP are higher than 6-layer HP, indicating that the existence of pores in laminates reduces the impact resistance of specimens. Figure 14c,d verify that COF can enhance the mechanical properties of laminates to resist the dynamic impact loading. The strain hardening is also shown to be sensitive to strain rate by comparing Figure 14b,c.

Experimental and computational results of max stress and max energy are illustrated in Table 6. The error between simulation results and experimental results is less than 9%, and the difference is small, which further verifies the reliability of the experiments. Also, it shows that the incorporation of COF does enhance the dynamic impact performance of the helicoidal composite laminates, which can absorb greater impact energy.

## 4. Conclusions

In order to explore the enhancement of the impact resistance of helicoidal PLA by continuous optical fiber (COF), PLA specimens with different layers and PLA specimens reinforced with COF were prepared respectively, and the specimens were compressed to different degrees. Then the stress-strain curves, maximum stress value, energy curves and dynamic compression deformation of 6-layer HP, 6-layer CHP, 6-layer COF-HP, 6-layer COF-CHP and 7-layer COF-CHP were analyzed. The results of the high-strain-rate dynamic impact loading test and finite element simulation show that the addition of COF can indeed improve the impact resistance of laminates in the case of pore elimination. The results show that the appropriate reduction in porosity can enhance the toughness of the specimen. When the porosity is reduced, the maximum stress of the continuous fiber-reinforced composite material is 4~7% higher than that of the pure PLA material. The strengthening mechanism is the fracture toughness of the bionic composite material being further improved after the introduction of the continuous fiber, so as to achieve the purpose of dissipating the external load energy. The mechanical properties of the laminates are enhanced.

## Figures and Tables

**Table 1:** Parameters for manufacturing conditions and process variables.

	Dimensions		Printing Parameters		Density *ρ* (kg/m^3^)	COF Doping (Y/N)
	*D* (mm)	*t* (mm)	Layer *T* (mm)	Nozzle *d* (mm)		
6-layer HP	16	12	0.4	1.4	1220	N
6-layer CHP	16	10	0.4	1.4	1464	N
6-layer COF-HP	16	12	0.4	1.4	1230	Y
7-layer COF-HP	16	14	0.4	1.4	1230	Y
8-layer COF-HP	16	16	0.4	1.4	1230	Y
6-layer COF-CHP	16	10	0.4	1.4	1476	Y
7-layer COF-CHP	16	10	0.4	1.4	1722	Y
8-layer COF-CHP	16	10	0.4	1.4	1968	Y

**Table 2:** Elastic and strength properties of COF-HP and HP.

Elastic Constants	Value (COF-HP/HP)	Elastic Constants	Value (COF-HP/HP)
Modulus in fiber direction *E*_1_ = *E*_2_ (GPa)	0.99/0.8	Longitudinal tensile strength *X*_t_ (MPa)	62.26/60.70
Transverse moduli *E*_3_ (GPa)	0.81/1.1	Longitudinal compressive strength *X*_c_ (MPa)	80/74
Shear moduli *G*_12_ (GPa)	0.35/0.3	Transverse tensile strength *Y*_t_ (MPa)	44.33/60.08
Shear moduli *G*_13_ = *G*_23_ (GPa)	0.31/0.44	Transverse compressive strength *Y*_c_ (MPa)	60/69
Poisson’s ratio *v*_12_	0.4/0.3	Shear strength *S*_12_ (MPa)	23/12
Poissons’ ratio *v*_13_ = *v*_23_	0.3/0.24	Shear strength *S*_13_ = *S*_23_ (MPa)	31.07/21
Density *ρ* (kg/m^3^)	1230/1220		

**Table 3:** Tensile testing of COF-PLA Composite material and PLA.

	Stress σ_max_ (MPa)	Strain ε
COF-filament	62.26	0.123
PLA-filament	60.7	0.148
COF-contact	44.32	0.110
PLA-contact	57.48	0.118

**Table 4:** Mechanical characteristics of specimens with the strain rate of 680 $s^{-1}$.

	*E_tan_* (GPa)	*σ_max_* (MPa)	*ε*
6-layer HP	1.08	104.84	0.059
6-layer CHP	0.88	109.92	0.065
6-layer COF-HP	0.79	78.07	0.072
6-layer COF-CHP	0.64	97.18	0.068
7-layer COF-CHP	1.26	108.10	0.075
8-layer COF-CHP	0.90	97.83	0.087

**Table 5:** Mechanical characteristics of specimens with the strain rate of 890 $s^{-1}$.

	*E_tan_* (GPa)	*σ_max_* (MPa)	*ε*
6-layer HP	0.81	110.63	0.067
6-layer CHP	0.63	113.00	0.065
6-layer COF-HP	0.68	80.79	0.091
6-layer COF-CHP	0.79	101.62	0.083
7-layer COF-CHP	1.32	118.10	0.075
8-layer COF-CHP	1.08	110.56	0.065

**Table 6:** Comparison between experimental and computational specimens subjected to max stress and max energy.

	Max Stress (MPa)		Max Energy (J)	
	Experiments	Computations	Experiments	Computations
6-layer HP (680 $s^{-1}$)	104.84	98.55	36.57	39.40
6-layer CHP (680 $s^{-1}$)	109.92	99.75	47.46	49.34
6-layer CHP (890 $s^{-1}$)	113.00	118.30	59.98	56.22
7-layer COF-CHP (890 $s^{-1}$)	118.10	118.70	70.14	71.05

## Data Availability

The data presented in this study are available on request from the corresponding author.

## References

[1] Sadeghi H., Davey K., Darvizeh R., Darvizeh A. (2019). A scaled framework for strain rate sensitive structures subjected to high rate impact loading. Int. J. Impact Eng..

[2] Walter T., Subhash G., Sankar B., Yen C. (2009). Damage modes in 3D glass fiber epoxy woven composites under high rate of impact loading. Compos. Part B Eng..

[3] Yamamoto N., de Villoria R.G., Wardle B.L. (2012). Electrical and thermal property enhancement of fiber-reinforced polymer laminate composites through controlled implementation of multi-walled carbon nanotubes. Compos. Sci. Technol..

[4] Bian Y., Chai H., Ye S., Xie H., Yao X., Cai Y. (2021). Compression and spallation properties of polyethylene terephthalate under plate impact loading. Int. J. Mech. Sci..

[5] Reddy T.S., Reddy P.R.S., Madhu V. (2017). Response of E-glass/epoxy and Dyneema^®^ composite laminates subjected to low and high velocity impact. Procedia Eng..

[6] Zhou J., Liu J., Zhang X., Yan Y., Jiang L., Mohagheghian I., Dear J., Charalambides M. (2019). Experimental and numerical investigation of high velocity soft impact loading on aircraft materials. Aerosp. Sci. Technol..

[7] Rivera J., Yaraghi N.A., Huang W., Gray D., Kisailus D. (2020). Modulation of impact energy dissipation in biomimetic helicoidal composites. J. Mater. Res. Technol..

[8] Wang J., Li N., Fu K., Li Y., Yang B. (2022). Nature-Mimic Tough Helicoidal Composites with Aligned Short Carbon Fibers by 3D Printing. Macromol. Mater. Eng..

[9] Yin S., Guo W., Wang H., Huang Y., Yang R., Hu Z., Chen D., Xu J., Ritchie R.O. (2021). Strong and tough bioinspired additive-manufactured dual-phase mechanical metamaterial composites. J. Mech. Phys. Solids.

[10] Yin S., Yang R., Huang Y., Guo W., Chen D., Zhang W., Ren M., Zhou Y., Xu J. (2021). Toughening mechanism of coelacanth-fish-inspired double-helicoidal composites. Compos. Sci. Technol..

[11] Zimmermann E.A., Gludovatz B., Schaible E., Dave N.K., Yang W., Meyers M.A., Ritchie R.O. (2013). Mechanical adaptability of the Bouligand-type structure in natural dermal armour. Nat. Commun..

[12] Zaheri A., Fenner J.S., Russell B.P., Restrepo D., Daly M., Wang D., Hayashi C., Meyers M.A., Zavattieri P.D., Espinosa H.D. (2018). Revealing the mechanics of helicoidal composites through additive manufacturing and beetle developmental stage analysis. Adv. Funct. Mater..

[13] Yang R., Zaheri A., Gao W., Hayashi C., Espinosa H.D. (2017). AFM identification of beetle exocuticle: Bouligand structure and nanofiber anisotropic elastic properties. Adv. Funct. Mater..

[14] Amorim L., Santos A., Nunes J., Dias G., Viana J. (2021). Quasi static mechanical study of vacuum bag infused bouligand inspired composites. Polym. Test..

[15] Liu Y., Shen J., Li Y., Ge X., Li Y. (2022). Enhanced high-strain-rate impact resistance of helicoidal composites by fused deposition modelling. Mech. Adv. Mater. Struct..

[16] Faruk O., Bledzki A.K., Fink H.-P., Sain M. (2012). Biocomposites reinforced with natural fibers: 2000–2010. Prog. Polym. Sci..

[17] Le Duigou A., Davies P., Baley C. (2011). Environmental impact analysis of the production of flax fibres to be used as composite material reinforcement. J. Biobased Mater. Bioenergy.

[18] Satyanarayana K.G., Arizaga G.G., Wypych F. (2009). Biodegradable composites based on lignocellulosic fibers—An overview. Prog. Polym. Sci..

[19] Eshkoor R., Ude A., Oshkovr S., Sulong A., Zulkifli R., Ariffin A., Azhari C. (2014). Failure mechanism of woven natural silk/epoxy rectangular composite tubes under axial quasi-static crushing test using trigger mechanism. Int. J. Impact Eng..

[20] Rezghi Maleki H., Hamedi M., Kubouchi M., Arao Y. (2019). Experimental investigation on drilling of natural flax fiber-reinforced composites. Mater. Manuf. Process..

[21] Zhu Y., Liu J., Liu D., Xu H., Yan C., Huang B., Hui D. (2017). Fiber path optimization based on a family of curves in composite laminate with a center hole. Compos. Part B Eng..

[22] Van Campen J.M., Kassapoglou C., Gürdal Z. (2012). Generating realistic laminate fiber angle distributions for optimal variable stiffness laminates. Compos. Part B Eng..

[23] Liu J.L., Mencattelli L., Zhi J., Chua P.Y., Tay T.-E., Tan V.B.C. (2022). Lightweight, Fiber-Damage-Resistant, and Healable Bio-Inspired Glass-Fiber Reinforced Polymer Laminate. Polymers.

[24] Zaretsky E., DeBotton G., Perl M. (2004). The response of a glass fibers reinforced epoxy composite to an impact loading. Int. J. Solids Struct..

[25] Ashrafi B., Guan J., Mirjalili V., Zhang Y., Chun L., Hubert P., Simard B., Kingston C.T., Bourne O., Johnston A. (2011). Enhancement of mechanical performance of epoxy/carbon fiber laminate composites using single-walled carbon nanotubes. Compos. Sci. Technol..

[26] Deniz M.E., Ozen M., Ozdemir O., Karakuzu R., Icten B.M. (2013). Environmental effect on fatigue life of glass–epoxy composite pipes subjected to impact loading. Compos. Part B Eng..

[27] Unterweger C., Duchoslav J., Stifter D., Fürst C. (2015). Characterization of carbon fiber surfaces and their impact on the mechanical properties of short carbon fiber reinforced polypropylene composites. Compos. Sci. Technol..

[28] Goh G.D., Dikshit V., Nagalingam A.P., Goh G.L., Agarwala S., Sing S.L., Wei J., Yeong W.Y. (2018). Characterization of mechanical properties and fracture mode of additively manufactured carbon fiber and glass fiber reinforced thermoplastics. Mater. Des..

[29] Huang Y., Meng X., Xie Y., Lv Z., Wan L., Cao J., Feng J. (2018). Friction spot welding of carbon fiber-reinforced polyetherimide laminate. Compos. Struct..

[30] Hu J., Yin S., Yu T., Xu J. (2018). Dynamic compressive behavior of woven flax-epoxy-laminated composites. Int. J. Impact Eng..

[31] Kousiatza C., Tzetzis D., Karalekas D. (2019). In-situ characterization of 3D printed continuous fiber reinforced composites: A methodological study using fiber Bragg grating sensors. Compos. Sci. Technol..

[32] Ouyang J., Chen X., Huangfu Z., Lu C., Huang D., Li Y. (2019). Application of distributed temperature sensing for cracking control of mass concrete. Constr. Build. Mater..

[33] Yan R., Wang Y., Luo P., Li Y., Lu X. (2022). Fused filament fabrication of continuous optic fiber reinforced polylactic acid composites. Rapid Prototyp. J..

[34] Li N., Link G., Jelonnek J. (2020). 3D microwave printing temperature control of continuous carbon fiber reinforced composites. Compos. Sci. Technol..

[35] Sarvestani H.Y., Akbarzadeh A., Niknam H., Hermenean K. (2018). 3D printed architected polymeric sandwich panels: Energy absorption and structural performance. Compos. Struct..

[36] Lei M., Hamel C.M., Yuan C., Lu H., Qi H.J. (2018). 3D printed two-dimensional periodic structures with tailored in-plane dynamic responses and fracture behaviors. Compos. Sci. Technol..

[37] Yin S., Chen H., Yang R., He Q., Chen D., Ye L., Mai Y.-W., Xu J., Ritchie R.O. (2020). Tough nature-inspired helicoidal composites with printing-induced voids. Cell Rep. Phys. Sci..

[38] Sharma A., Mishra R., Jain S., Padhee S.S., Agnihotri P.K. (2018). Deformation behavior of single and multi-layered materials under impact loading. Thin-Walled Struct..

[39] Gray III G., Blumenthal W.R. (2000). Split-Hopkinson pressure bar testing of soft materials. ASM Handb..

[40] Kolsky H. (1949). An investigation of the mechanical properties of materials at very high rates of loading. Proc. Phys. Soc. Sect. B.

